# Effects of *Bifidobacterium Breve* Feeding Strategy and Delivery Modes on Experimental Allergic Rhinitis Mice

**DOI:** 10.1371/journal.pone.0140018

**Published:** 2015-10-07

**Authors:** Jian-jun Ren, Zhao Yu, Feng-Ling Yang, Dan Lv, Shi Hung, Jie Zhang, Ping Lin, Shi-Xi Liu, Nan Zhang, Claus Bachert

**Affiliations:** 1 Department of Oto-Rhino-Laryngology West China Hospital, West China Medical School, Sichuan University, Chengdu, China; 2 Upper Airways Research Laboratory, West China Hospital, Sichuan University, Chengdu, China; 3 Upper Airways Research Laboratory (URL), Department of Oto-Rhino-Laryngology, Ghent University, Ghent, Belgium; Wayne State University, UNITED STATES

## Abstract

**Background:**

Different delivery modes may affect the susceptibility to allergic diseases. It is still unknown whether early intervention with probiotics would counteract this effect.

**Objectives:**

The effect of different delivery modes on immune status and nasal symptoms was investigated on established allergic rhinitis (AR) mouse model. In addition, the immunoregulatory effects and mechanisms of different feeding manners with *Bifidobacterium breve*(*B*. *breve*) were examined.

**Methods:**

Live lyophilized *B*. *breve* was orally administered to BALB/c mice born via vaginal delivery(VD) or cesarean delivery (CD) for 8 consecutive weeks, after which they were sensitized by ovalbumin(OVA) to establish experimental AR. Nasal symptoms, serum immunoglobulins, cytokines, splenic percentages of CD4^+^CD25^+^Foxp3^+^ regulatory T(Treg) cells and nasal eosinophil infiltration were evaluated.

**Results:**

Compared with VD mice, mice delivered via CD demonstrated more serious nasal symptoms, higher concentrations of OVA-specific immunoglobulin (Ig) E, more nasal eosinophils and lower percentages of splenic CD4^+^CD25^+^Foxp3^+^Treg cells after establishing experimental AR. These parameters were reversed by administering *B*. *breve*s hortly after birth. However, the effect of *B*. *breve* did not differ between different delivery modes.

**Conclusion:**

CD aggravates the nasal symptoms of AR mice compared to VD. This is the first report that oral administration of *B*. *breve* shortly after birth can significantly alleviate the symptoms of AR mice born via both deliveries, probably via activation of the regulatory capacity of CD4^+^CD25^+^Foxp3^+^Treg cells.

## Introduction

Allergic rhinitis (AR) is a chronic inflammatory condition of the upper airways, characterized by symptoms such as nasal itching, sneezing, rhinorrhea and elevated serum immunoglobulin E (IgE) levels. In recent decades, the incidence of AR has increased significantly worldwide, which may be related to diet structure changes, popular use of antibiotics and other factors involving microbes. According to the “hygiene hypothesis”, the changes in microbial burden early in life would lead to increased susceptibility to allergy in later life[1].

The homeostasis of early gut microbiotais considered essential for the maturation and maintenance of the host immune system[2]. Germ-free mice at neonatal age are not able to induce oral tolerance. When reconstituted with probiotics such as *Bifidobacterium (B*.*)*, the susceptibility of T helper type 2 (Th2) responses to oral tolerance induction is restored to a level comparable with specific pathogen-free mice[3]. Furthermore, in infants who are predisposed to develop allergies (later in life), the composition of the gut flora is quite different from infants who are not predisposed, even before any clinical manifestations of atopy are observed[4]. Multiple factors can affect the early microbial establishment, such as genetic background, gestational age, modes of delivery and feeding type, as well as antibiotic therapy at an early age[5].

The first exposure to environmental microbiota occurs during the initial hours of delivery. The mode of delivery influences the establishment of microbiota and its subsequent roles in immune regulation. Infants born via vaginal delivery (VD)are predominantly colonized with *Lactobacillus*, *Prevotella* or *Sneathia* species in the intestine, resembling their mother’s vaginal microbiota; infants born via cesarean delivery(CD) acquire bacterial communities from the maternal skin surface, dominated by *Staphylococcus*, *Corynebacterium* and *Propionibacterium* species[6]. In later life, the persistent changes of intestinal microbiota induced by CD will increase the risk of atopic diseases such as asthma, AR, atopic dermatitis and food allergy[7]. However, the precise mechanisms of increased incidence of AR caused by CD are not yet understood.

Probiotics, defined as “live microorganisms that confer a health benefit on the host when consumed in adequate numbers”, are major components of the gut flora[8]. Currently, evidence is accumulating that some probiotic strains have shown potential effectiveness in preventing atopic diseases via both innate immunity and adaptive immunity[9].*B*. *breve*, which is one strain of *Bifidobacteria* originally isolated from healthy infant feces, has been reported to successfully prevent or modify some immune disturbance caused by atopy[10,11]. In a previous study, the immunoregulatory capacities of *B*.*breve*, *Lactobacillus GG* and mixed probiotics (*B*.*breve* and *Lactobacillus*) were compared, by measuring the concentration of IgE in serum and the splenic percentage of CD4^+^CD25^+^regulatory T (Treg) cells in BALB/c mice with established AR (AR) mice. This study showed that *B*.*breve* was one of the most potent anti-allergic strains (unpublished data). However, the exact regulatory mechanisms by which *B*. *breve* regulates the immunological status disturbed by CD in infants with AR still need to be studied.

In the present study, the effects of CD on nasal symptoms and immune status of ovalbumin (OVA)-sensitized BALB/c AR mice were examined. As AR is generally associated with an imbalance of Th1 and Th2 cells and with a dysfunction of Treg cells, the levels of Th1- and Th2-associated cytokines, total IgE and OVA-specific IgE were measured in serum as well as splenic CD4^+^CD25^+^Foxp3^+^Treg cell percentages. Furthermore, by administration of *B*. *breve* via different feeding manners to the pups shortly after CD, it was analyzed whether *B*. *breve* has inconsistent immunomodulating capacities in experimental AR mice.

## Materials and methods

### Animals

Female and male BALB/c mice in specific pathogen-free conditions, aged 6-8weeks, were obtained from the Dashuo Laboratory Animal Co., Ltd (Chengdu, Sichuan, China). All animals were kept in plastic cages and allowed free access to food and water in an environment-controlled room(24±1°C, 55% humidity, 12/12h day-night light cycles). Generally, 3–5 female mice were kept with 1 male mouse within one cage for mating. When a sperm plug was observed in the vagina, the female mouse was considered pregnant and reared in a separate cage until VD or CD. After birth, only female pups were used for further research. The female mice were randomly assigned to 7 groups (n = 12–15 per group) and received different experimental operations:

1. VD (VD)
2. CD (CD)
3. experimental AR establishment after VD (VD+AR)
4. experimental AR establishment after CD (CD+AR)
5. *B*. *breve* feeding and experimental AR establishment after VD (VD+B.+AR)
6. *B*. *breve* feeding and experimental AR establishment after CD (CD+B.+AR)
7. *B*. *breve* feeding immediately after CDand thereafter experimental AR establishment (CD+B._-immediate_ +AR)

All procedures were performed according to the guide for the care and use of laboratory animals and approved by the Laboratory Animal Ethics Committee of Sichuan University.

### Cesarean Section

The procedures of cesarean section were followed as previously described by Murphy[12]. Briefly, on day 20 of gestation(day 1: sperm plug observed; day 20: expected day of delivery), the mother was killed by cervical dislocation and disinfected with ethanol. The abdominal wall was cut open, the uterus was carefully sectioned and the pups were gently squeezed out. The surgical procedures were performed under sterile conditions.

### Probiotic Treatment

**Fig 1:**
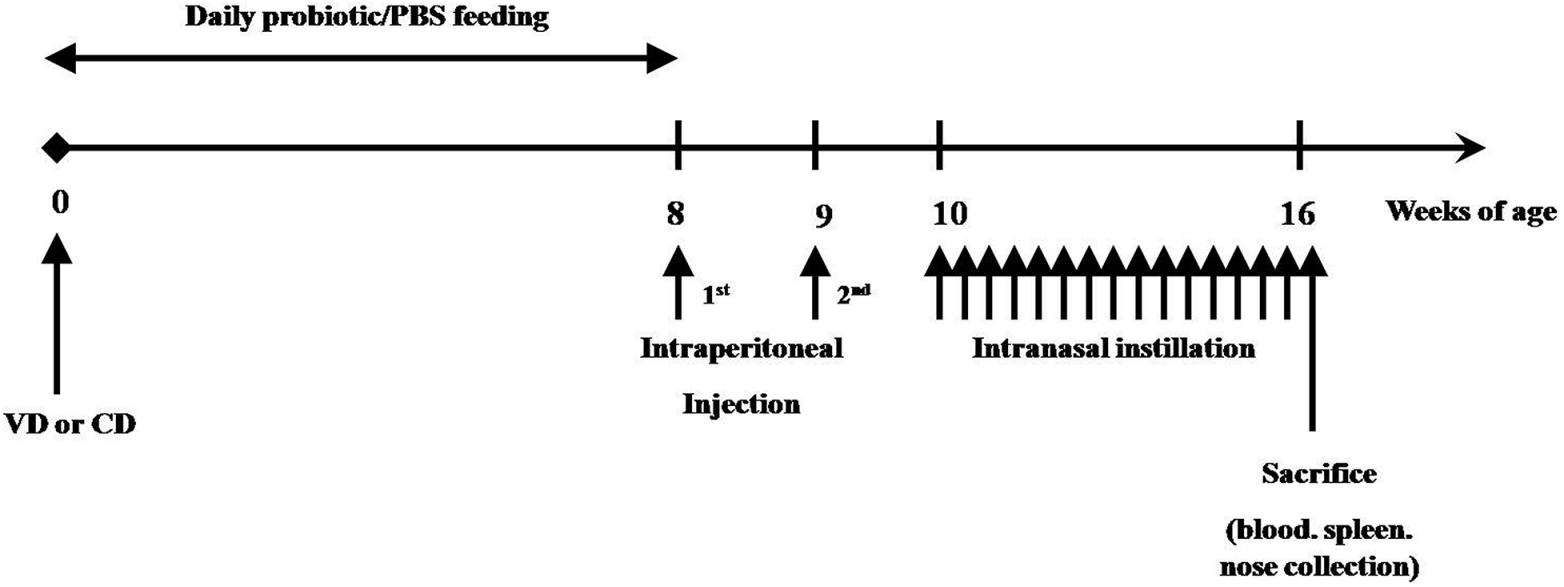
The experimental protocol in the present study. Mice were given *B*. *breve* daily from week 0 to week 7 short after birth via VD or CD, then they were sensitized from week 8 to week 15 to establish experimental AR. At week 16, mice were sacrificed.

Live lyophilized *B*. *breve* was provided by Jiao Da Onlly Lmt., Co.(Shanghai, China). Starting at the first day afterVD or CD, pups were given*B*. *breve* by gavage. In the CD+B.-1^st^bite+AR group, pups were orally given *B*. *breve* before breastfeeding or any other food shortly after CD. The concentration of *B*. *breve*was10^9^ colony-forming units(CFU)/ml distilled in phosphate buffered saline (PBS), and the volume of daily administration increased according to the age of the offspring:25μl at 0-1weeks, 50μl at 2–3 weeks, 75μl at 4–5 weeks and 100μl at 6–7 weeks after birth. Pups in control groups were given the same volume of PBS. The oral administration was carried out once a day for 8 consecutive weeks (Fig 1).

### Experimental AR Establishment and Nasal Symptoms Evaluation

After 8 weeks of *B*.*breve* administration, mice were sensitized with OVA(Sigma, A5503 St. Louis USA) to establish experimental AR. Briefly, mice were primarily sensitized twice by intraperitoneal injection of 200μl PBS containing 20μg OVA at week 8 and 9 after birth. At week10, they were intranasally instilled with200μg OVA distilled in 20μl PBS once daily for 6 consecutive weeks (Fig 1). Mice in control groups were administered the same volume of PBS.

To evaluate the nasal symptoms, mice were placed in an observation cage, and the frequency of sneezing and nasal rubbing was counted during10minutes, within 30minutes after intranasal instillation. The evaluation of nasal symptoms was carried out once a week during the period of nasal challenge.

At week16, mice were anesthetized by the mixture of ketamine and xylazine hydrochloride to collect blood from ophthalmic vein. After then, they were sacrificed to collect noses and spleens.

### Measurement of Serum total IgE and OVA-specific IgE

Total IgE(eBioscience, 88390107, San Diego USA)as well as OVA-specific IgE(Biolegend, 439807, California USA) levels in serum were measured by enzyme-linked immunosorbent assay(ELISA) according to the manufacturer’s instructions. For the measurement of total IgE, samples were diluted 1:100 in deionized water, while 1:1–1:6 dilutions were used for OVA-specific IgE. Absorbance values were read at 450nm on a Bio-Rad 680 microplate reader. Total immunoglobulin levels were calculated with reference to a standard. The minimal detection concentration was 4ng/ml for total IgE and 20.7pg/ml for OVA-specific IgE.

### Serum Cytokine Analysis

Interleukin (IL)-4, IL-5and interferon (IFN)-γ levels in serum were measured by using Luminex kits(R&D, LUM000, Minneapolis USA) according to the manufacturer’s instructions and analyzed using a Luminex100 instrument. Samples were diluted1:2 in deionized water.

### Splenocyte Preparation and Flow Cytometry Analysis of Treg cells

After removal of the spleen, half of the spleen was gently grinded and filtered through a 40μm filter to obtain cell suspensions. Approximately 1x10^6^splenocytes suspended in 1 ml PBS were stained with FITC-labelled anti-mouse CD4 and PE-labelled anti-mouse CD25 for 30 minutes at 4°C. After permeabilization and fixation, the splenocytes were intracellularly stained with APC-labelled anti-mouse Foxp3 following the manufacturer’s instructions(all antibodies from eBioscience, E09667-1636, San Diego USA). Treg cells, defined as CD4^+^CD25^+^Foxp3^+^ cells, were detected in the splenocytes by flow cytometry (BD, FACSAria^TM^) and data were analyzed by FlowJo 7.6 software.

### Histological Observation in Nasal Mucosa

When mice were sacrificed at week 16, their noses were collected for histological observation. The head was removed and the lower jaw, skin and soft tissue were discarded. The noses were decalcified in 10% nitric acid solution for a total of 5 days before regular paraffin embedding. Serial sections were cut at the level of middle turbinate and inferior turbinate and stained with hematoxylin and eosin for microscopic analysis of the morphology of the nasal mucosa. Investigator was blinded for the treatment groups and calculated the average numbers of eosinophils in 10 high-magnification (×400) fields in each group.

### Statistical Analysis

Statistical analysis was performed using SPSS version 17.0. Data with normal distribution and homogeneity of variance were analyzed using the Student—Newman—Keuls and Dunnett-t methods for pairwise comparison; data without normal distribution and homogeneity of variance were analyzed using a Kruskal-Wallis H rank sum test. A p-value<0.05 was considered statistically significant.

## Results

### Experimental AR Establishment

Experimental AR of mice was established by sensitization with OVA (Sigma, A5503 St. Louis USA)following8 weeks of *B*. *breve* administration. As shown in Fig 1, mice were primarily sensitized twice by intraperitoneal injection of 200μl PBS containing 20μg OVA at week 8 and 9 after birth. At week10, they were intranasally instilled with2 00μg OVA distilled in 20μl PBS once daily for 6 consecutive weeks. Mice in control groups were administered the same volume of PBS.

### Effects of Nasal Rubbing and Sneezing

**Fig 2:**
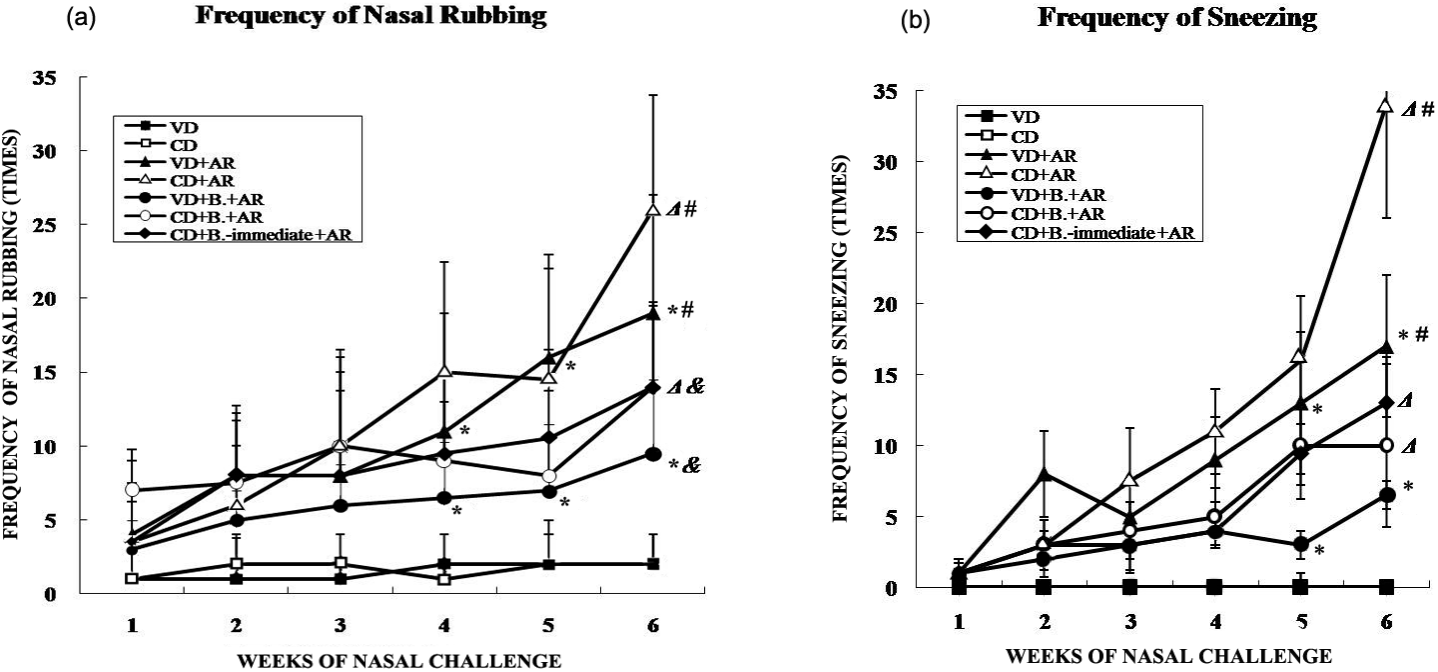
Effects of Nasal Rubbing and Sneezing. Frequency of (a)nasal rubbing and (b)sneezing during the period of nasal challenge among 7 groups. *: VD+B.+AR group compared with VD+AR group, p<0.05; Δ: CD+B.+AR group and CD+B.-immediate+AR group compared with CD+AR group, p<0.05; &: CD+B.+AR group and CD+B.-immediate+AR group compared with VD+B.+AR group, p<0.05; #: CD+AR group compared with VD+AR group, p<0.05.

Fig 2 ademonstrates the results of nasal rubbing in the 7 groups during the period of nasal challenge. There was a statistically significant difference in nasal rubbing frequency between the VD+AR group and CD+AR group after 6 weeks of nasal challenge. Oral administration of *B*. *breve* significantly decreased the frequency of nasal rubbing in both VD- and CD-born AR mice, especially at week5 of nasal challenge. However, *B*. *breve* did not cause any differences in inhibition of nasal rubbing between mice born by VD and mice born by CD with established AR. Similarly, there were also no obvious differences in nasal rubbing between the CD+B.+AR group and the CD+B._-immediate_+AR group.

As shown in Fig 2b, the sneezing frequency was significantly increased in the CD+AR group compared to the VD+AR group at week 5 and 6 of nasal challenge. Oral administration of *B*. *breve* shortly after birth significantly suppressed the sneezing frequency in both VD- and CD-born AR mice at week5 of nasal challenge. However, *B*. *breve* did not show any different degree of inhibition between VD- and CD-born AR mice, and there was also no significant difference in sneezing suppression when *B*. *breve* was given first immediately or not to CD-born AR mice.

### Serum IgE Levels

**Fig 3:**
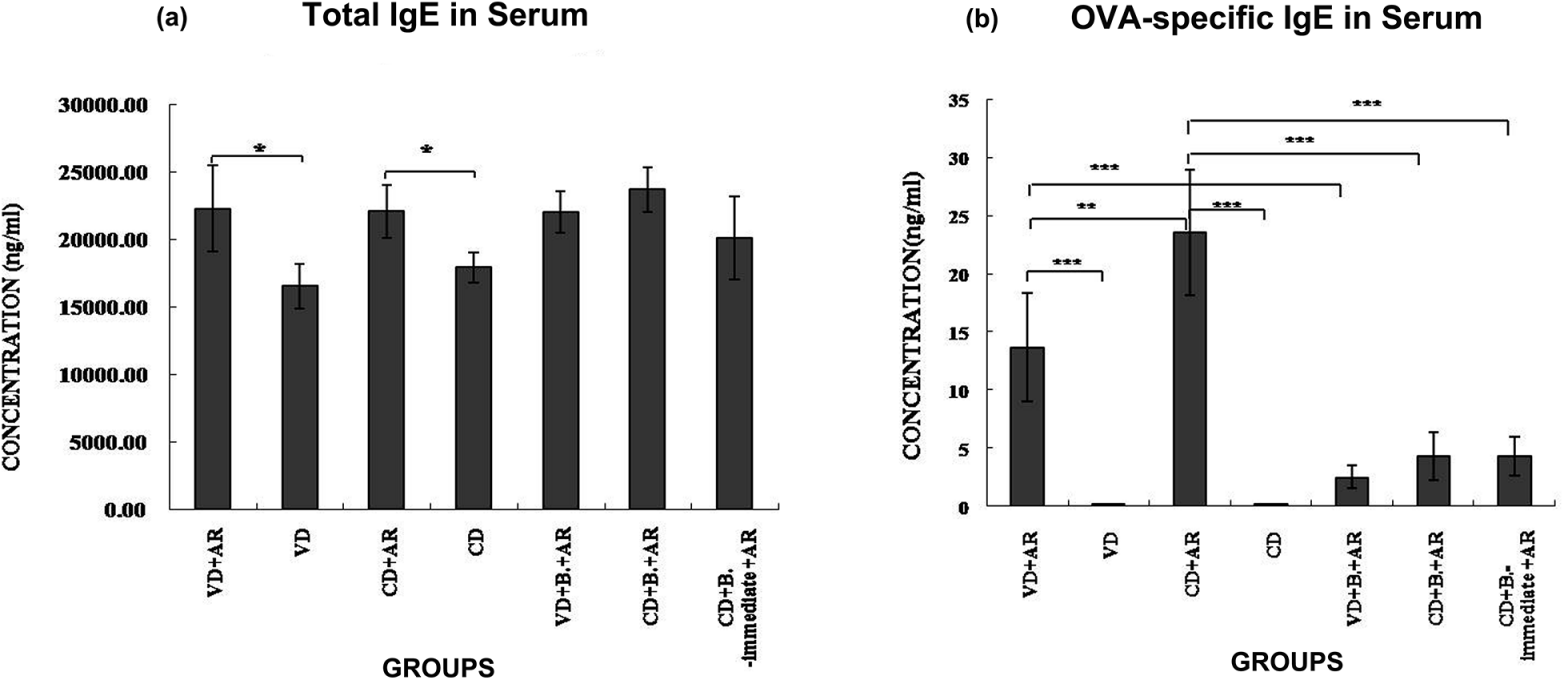
Serum IgE Levels. The concentration of (a)total IgE and (b)OVA-sIgEin serum among 7groups.*: p<0.05, **:p<0.01, ***:p<0.001.

Compared with the corresponding negative control groups, the concentration of total IgE was significantly higher in VD+AR and CD+AR groups (Fig 3a). Administration of *B*. *breve* did not affect total IgE levels of mice with AR born by VD or CD.

The serum OVA-specific IgE concentration in both the VD+AR group and the CD+AR group was significantly higher compared to the concentration in the corresponding negative group (Fig 3b). Furthermore, the OVA-specific IgE concentration was significantly higher in the CD+AR group compared with the VD+AR group. After giving *B*. *breve* orally for 8 weeks in the VD+AR group and the CD+AR group, the concentration of serum OVA-specific IgE was significantly decreased. After treatment with the *B*.*breve*, the serum OVA-specific IgE levels in the VD+B+AR group and CD+B+AR/ CD+B._-immediate_+AR group decreased by 5.7 times and 5.3 times respectively, compared to untreated rhinitis groups. No significant differences were observed between the three groups fed with *B*. *breve*.

### Concentrations of Serum Cytokines

No differences in serum levels of IL–4, IL–5 and IFN-γ were observed between the 7 groups (data not shown).

### Percentage of CD4^+^CD25^+^Foxp3^+^Treg Cells in Spleen

**Fig 4:**
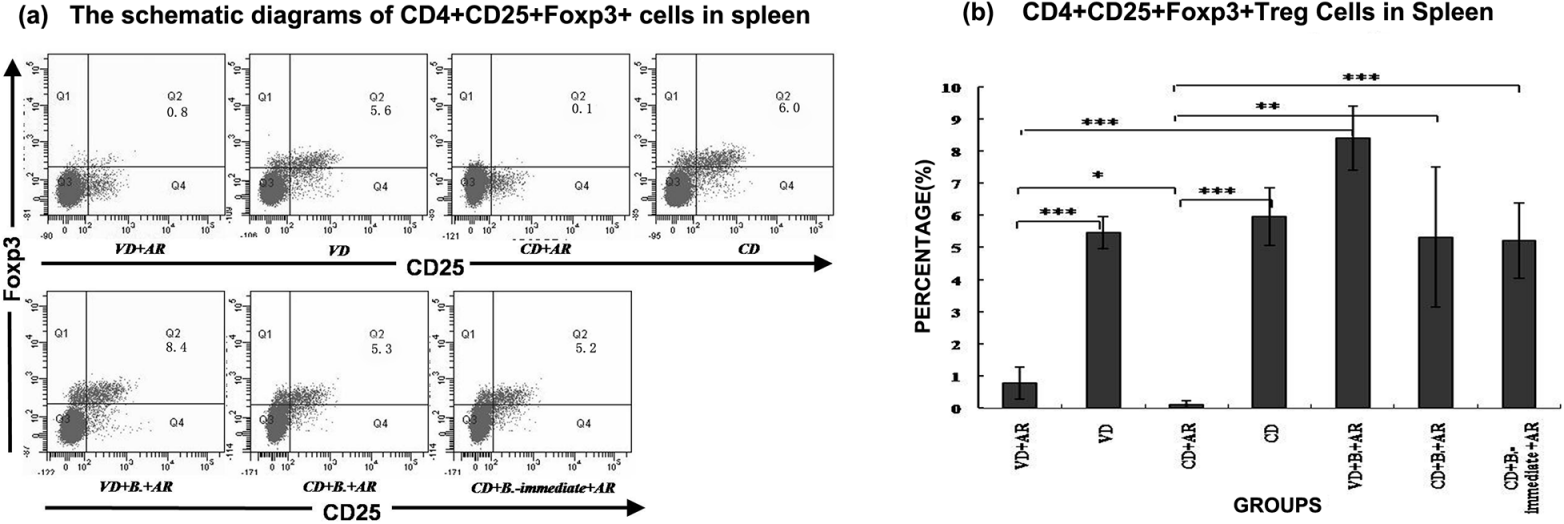
Percentage of CD4^+^CD25^+^Foxp3^+^Treg Cells in Spleen. The (a)schematic diagrams and (b)percentage ofCD4+CD25+Foxp3+ cells in spleen among 7 groups. Q2: CD4+CD25+Foxp3+ cells. Q2: CD4+CD25+Foxp3+ cells. *: p<0.05, **:p<0.01, ***:p<0.001.

The percentage of CD4^+^CD25^+^Foxp3^+^Treg cells was significantly decreased in mice with established AR compared with the corresponding negative groups(Fig 4a and 4b). The percentage of Treg cells was significantly lower in the CD+AR group when compared with the VD+AR group. After the mice were fed with *B*. *breve*, the percentage of Treg cells was significantly increased in VD- and CD-born mice with AR, by 10.8 times and 48.9times higher than the untreated group respectively. No significant differences were observed between the three groups with *B*. *breve* administration.

### Histological Examination of Nasal Mucosa

**Fig 5:**
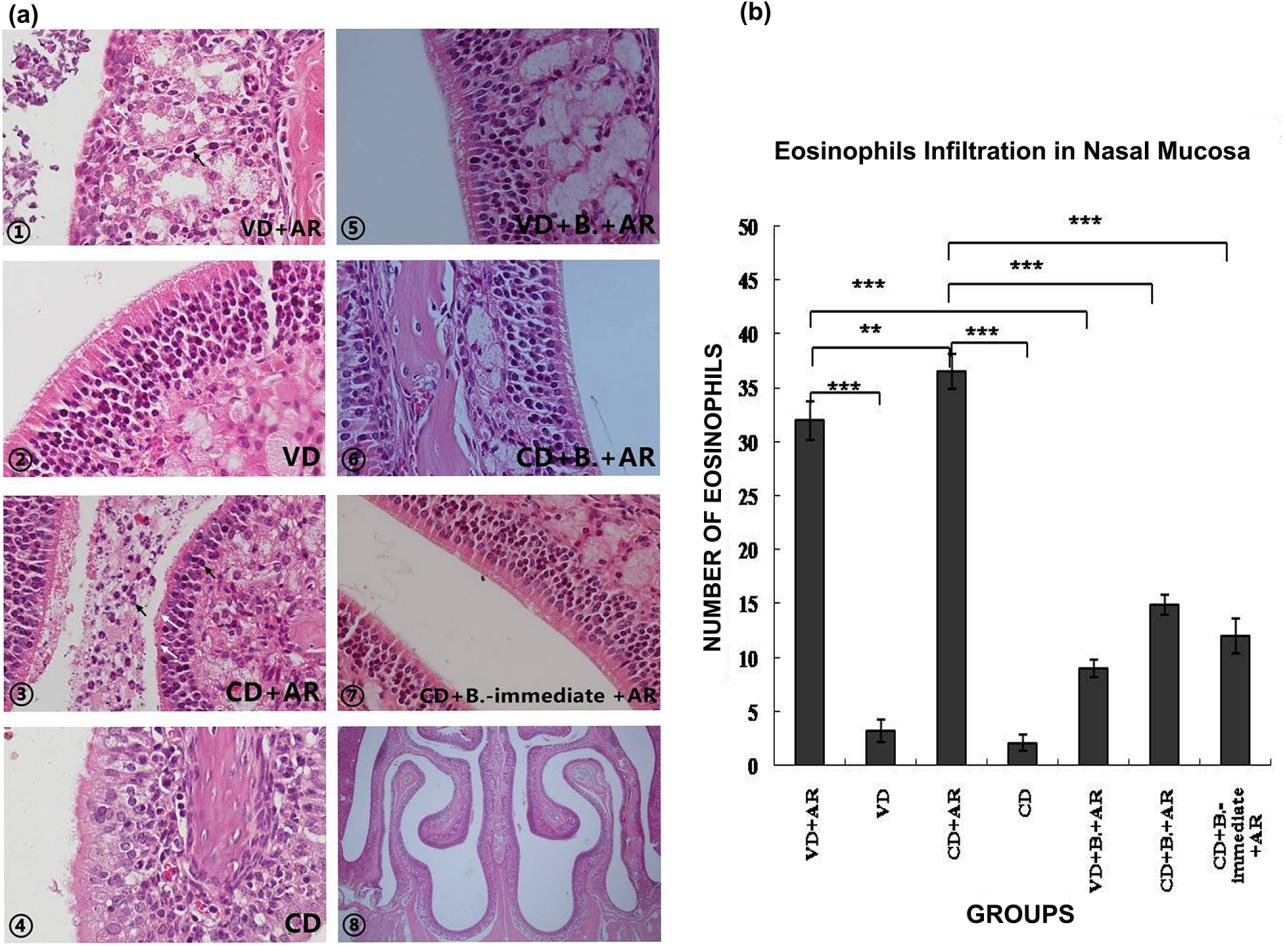
Histological Examination of Nasal Mucosa. (a)①-⑦:The histological characters of nasal mucosa among 7 groups, ×400, ⑧: The gross morphology of the nasal cavity of BALB/c mice, ×40. Black arrow: eosinophil infiltration; White arrow: detached cilia. (b)The numbers of eosinophils infiltrated in nasal mucosa among 7 groups.**:p<0.01, ***:p<0.001.

More eosinophils were infiltrated in the nasal mucosa and submucosal tissue of VD- and CD-born AR mice compared with non-AR mice, and the cilia were also detached (Fig 5a and 5b). After administration of *B*. *breve*, the impaired cilia were observed recovered, and the eosinophil infiltration of VD+B+AR group and CD+B+AR group was decreased by approximately 3.6 times and 2.5 times, compared to VD+AR group and CD+AR group respectivly. The eosinophil infiltrationin both the VD+AR group and the CD+AR group was increased compared with the VD group and CD group respectively, and the number of eosinophils in the CD+AR group was significantly higher than in the VD+AR group.

## Discussion

In the present study, the effects of *B*. *breve*on nasal symptoms and immune status in VD- or CD-born mice with experimental AR were investigated. Compared with VD mice after establishing AR, mice delivered by CD demonstrated higher concentrations of serum OVA-specific IgE, more eosinophil infiltration in the nasal mucosa and lower percentages of splenic CD4^+^CD25^+^Foxp3^+^Treg cells, and consequently, more serious nasal symptoms. By giving oral administration of *B*. *breve* shortly after birth for a total of 8 weeks, the nasal symptoms of AR ameliorated. Simultaneously, the altered immune status was re-modulated in AR mice, irrespective of delivery modes. However, mice fed with *B*. *breve* did not show any differences in Th1- and Th2-associated cytokines in serum. In addition, there was also no significant difference in the effects of different feeding manners (feeding first immediately after born or feeding after breastfeeding) of *B*. *breve* to CD mice after establishing AR. These results may indicate that *B*. *breve* affects the immune status of mice with experimental AR via a mechanism involvingCD4^+^CD25^+^Foxp3^+^Treg cells.

Interestingly, some studies focused on using bispecific antibodies [13]and antibody multimers[14] in the prevention and treatment of autoimmune diseases. They also found that treatment with bispecific antibodies or antibody multimers could produce effective suppression of established animal autoimmune disease modal, accompanied by expansion in the population of Tregs, and down-modulation of pathogenic antibody responses, which were somewhat similar to our research.

AR is a respiratory disease of the upper airways characterized by high concentrations of serum allergen-specific IgE, infiltration of inflammatory cells in the nasal mucosa and release of several inflammatory cytokines. Previous studies have suggested that the pathogenesis of allergic diseases is mainly associated with the imbalance of Th1/Th2 cells. In 1995, Sakaguchi[15]reported a new class of T cells, called “Treg cells”, which were demonstrated to play an important role in the development of immune tolerance and in the pathogenesis of allergic diseases[16,17]. Treg cells that express the lineage-specific transcription factor Foxp3 and Tr1-like cells that produce IL–10 comprise the major regulatory populations. Foxp3 acts as a master switch gene for CD4^+^CD25^+^Treg cell development and function, and a mutation in Foxp3 leads to hyper-IgE, eosinophilia and dysregulated Th1 and Th2 cytokine production[18]. Furthermore, some *Bifidobacteria* and lactic acid bacteria have been reported to induce immunoregulatory responses and attenuate both Th1 and Th2 responses by Treg cells, which in turn ameliorated the severity of some allergic diseases[19]. However, other studies demonstrated that probiotics could significantly decrease OVA-specific IgE production in OVA-immunized mice via an IFN-γ-independent pathway mediated by Treg cells. CD4^+^Foxp3^+^Treg cells could affect atopic patients by unknown factors, which were unrelated to Th1/Th2 markers[20]. Luping Zhu et al[21]also observed that oral administration of lysed *Enterococcus faecalis*FK–23, a probiotic product of *Enterococcus faecalis*, to BALB/c mice alleviated nasal symptoms, reduced nasal eosinophil infiltration and increased the percentage of CD4^+^CD25^+^Treg cells in experimental AR, without regulating serum and splenocyte culture supernatant levels of IL–4, IFN-γ and OVA-specific IgE. Therefore, Ozdemir et al concluded that, except for inhibition of Th1 and Th2-type cytokines, Treg cells could potently suppress IgE production and directly or indirectly suppress the activity of effector cells of allergic inflammation[17]. The above observations were partly consistent with the results from this study, in which the decreased serum OVA-specific IgE, nasal eosinophil infiltration and increased percentages of splenic CD4^+^CD25^+^Foxp3^+^Treg cells by *B*. *breve* were also observed. However, no effects were seen on systemic IL–4, IL–5 and IFN-γ concentrations. It may be hypothesized that *B*. *breve* increased the percentage of CD4^+^CD25^+^Foxp3^+^Treg cells in the spleen, which might decrease serum allergen-specific IgE and local eosinophils infiltration. However, further investigations are needed to demonstrate whether the mechanism of reducing OVA-specific IgE by *B*. *breve* occurs via CD4^+^CD25^+^Foxp3^+^Treg cells, independent of regulating the Th1/Th2 balance.

By combining the administration of *B*. *breve* with non-digestible oligosaccharides in a chronic asthma murine model, Sagar et al[22] found that this specific combination was beneficial for allergic asthma, possibly via the induction of CD4^+^CD25^+^Foxp3^+^Treg cell response. Hougee et al[11] compared the effects of several strains of probiotics on OVA-induced allergic asthma and *B*. *breve* M-16V was identified as the most potent anti-allergic strain. Furthermore, several other studies combined *B*. *breve* with probiotics or prebiotics to evaluate the efficacy on a given kind of cell or allergic disease[23,24,25,26]. These studies indicated that *B*. *breve* may play an important role in the pathogenesis and severity of allergic diseases. This study therefore investigated how *B*. *breve* individually could affect a murine model with established AR.

Ashour et al[27,28] established mice models immunized with OVA subcutaneously, and showed that B-cell expansion was required for antigen presenting B cells to induce the generation of Treg andthus the induction of T-cell tolerance, which indicated that B-cell played an important role in T cell responses. However, in our unpublished study, we have investigated the dose-response relationship of *B*.*breve* on a murine allergic rhinitis model, presenting results of decreased IL–4 and OVA-specific IgE levels and increased CD4+CD25+ Tregs in the spleen at a dose of 10^9^CFU.*B*. *breve* installed a Th1/Th2 equilibrium mainly through the inhibition of the Th2 reponse, without promoting a Th1 response. Thus, we concluded that *B*.*breve* exerted its anti-allergic effects by inhibiting Th2 immune responses and enhancing CD4+CD25+ Treg activity in that study. However, in the present study, no evidence was found that *B*.*breve* had the capacity to regulate the Th1/Th2 balance, which was partly contradictory to the previous findings and our unpublished study. We hypothesize that the conflicting conclusions might be due to different combinations (*B*.*breve* combined with probiotics, prebiotics or synbiotics)used, different kinds of allergic diseases, or selections of different study populations with possibly underlying discrepant immune status, which deserves more in-depth research.

In recent years, with the increasing prevalence of CD in many areas of the world, an epidemic of allergic diseases has been postulated[29]. A number of clinical prospective studies as well as systematic reviews demonstrated that CD influences the development of some allergic diseases, such as atopic asthma, atopic dermatitis, food allergy and AR[7,30]. However, only few studies focused on the immune mechanisms of how CD affects allergic diseases, although several studies have reported the close relation withthe composition and populations of intestinal microbiota, especially commensal bacteria[31]. In the present study, more serious symptoms and a more deviated immune status were observed in AR mice born via CD, compared with VD mice. Our observations suggest that CD aggravates AR symptoms by elevating the production of allergen-specific IgE, increasing local inflammatory cell infiltration and suppressing CD4^+^CD25^+^Foxp3^+^Treg cells. Moreover, we investigated how *B*. *breve* regulated the immune status of CD mice after being established with AR, since Mikael Kuitunen et al[32]observed a protective effect of probiotic mixture and prebiotic on allergic diseases in CD children in a randomized, double-blinded, placebo-controlled clinical study. We have also demonstrated that oral administration of *B*.*breve* shortly after CD significantly alleviated nasal symptoms in mice with AR, and the strength was almost equal to that in VD mice. Most current literature has suggested that the environment of a normal fetus is sterile, and microbiota begin to colonize rapidly during birth, which emphasizes the powerful effects of delivery modes on the composition of the microflora in the gastrointestinal tract of newborns. Nevertheless, some recent studies demonstrated that the microbial community has already dwelled in the fetus before birth, as bacteria or bacterial ribosomal DNA was detected in meconium or amniotic fluid[29]. In the present study, there was no significant difference whether *B*. *breve* was orally administered to cesarean-delivered mice immediately after birth or later. However, there was limited information concerning the intestinal microbiome in this study, which restricted the possibility of an in-depth exploration of the effects of *B*.*breve* in the gut.

## Conclusions

This study showed that the nasal symptoms (nasal rubbing and sneezing) of cesarean delivered mice with AR were more severe than vaginal delivered mice. The oral administration of *B*. *breve* shortly after birth for 8 consecutive weeks, whether immediately fed after birth or not, could significantly alleviate the nasal symptoms of CD mice with AR. The regulatory capacity did not vary between the delivery modes; *B*. *breve* increased the number of CD4^+^CD25^+^Foxp3^+^Treg cells, which might suppress allergen-specific IgE production and local mucosal infiltration of eosinophils.

## Abbreviations

AR: allergic rhinitis
*B. (breve)*: *Bifidobacterium breve*
CD: cesarean delivery/cesarean delivered
CFU: colony-forming unit
IFN: interferon
IgE: immunoglobulin E
IL: interleukin
OVA: ovalbumin
PBS: phosphate buffered saline
Th1/2: T helper type 1/2
Treg cell: regulatory T cell
VD: vaginal delivery/vaginal delivered
